# Translation, validity, and reliability of the Thai de Morton Mobility Index in patients following hip surgery

**DOI:** 10.1016/j.ijnss.2025.04.003

**Published:** 2025-04-15

**Authors:** Chanokporn Jitpanya, Surachai Maninet, Chanipa Yoryuenyong

**Affiliations:** a Faculty of Nursing, Suan Dusit University, Bangkok, Thailand; b Faculty of Nursing, Ubon Ratchathani University, Ubon Ratchathani, Thailand; c Kuakarun Faculty of Nursing, Navamindradhiraj University, Bangkok, Thailand

**Keywords:** Hip fractures, Patients, Recovery of function, Reliability, Validity

## Abstract

**Objective:**

This study aimed to translate the de Morton Mobility Index (DEMMI) into Thai and assess its measurement properties.

**Methods:**

The de Morton Mobility Index (DEMMI) was translated into Thai using a cross-cultural translation method. A cross-sectional study was conducted in four public hospitals in Thailand between January and March 2023. A total of 260 patients were recruited from outpatient clinics. Convergent and known-group validity were evaluated through hypothesis testing. Construct validity was examined using confirmatory factor analysis. Reliability was assessed using Cronbach’s α coefficient. We also employed the Rasch analysis to validate validity and person reliability.

**Results:**

Content validity was high (S-CVI = 0.96, I-CVI range: 0.80–1.00). Strong convergent validity was observed, with a significant correlation (*r* = 0.761, *P* < 0.001) between the Thai DEMMI and the Parker Mobility Scale (PMS). Known-group validity was evident, demonstrating differences in scores across various patient groups. A confirmatory factor analysis supported the hypothesized factor structure of the Thai DEMMI with good fit indices: *χ*^2^ (*df* = 4) = 5.101, *P* = 0.2771; *χ*^2^/*df* = 1.275, RMSEA = 0.033; CFI = 0.998; TLI = 0.995; SRMR = 0.016. The Thai DEMMI exhibited high internal consistency (Cronbach’s α = 0.88). Rasch analysis revealed good person reliability (0.91) and acceptable information-weighted fit means square statistic (0.73–1.06). However, most items showed good fit based on the outlier-sensitive fit means square statistics (Outfit MNSQ), one exhibited a high Outfit MNSQ value of 29.94, suggesting a potential misfit.

**Conclusion:**

This study demonstrated the acceptable validity and reliability of the Thai DEMMI. Further evaluation of its responsiveness to change is still recommended.

**What is known?**

• The de Morton Mobility Index (DEMMI) is a well-established tool developed in Western contexts to assess mobility in older adults.
• It is a unidimensional measure with strong measurement properties.
• The DEMMI is a performance-based bedside assessment that can be completed within 10 min without requiring special materials.

**What is new?**

• The Thai DEMMI is valid and reliable, providing a valuable tool for clinicians and researchers in Thailand to enhance mobility assessment and care planning for Thai patients.

## 1 Introduction

Hip fracture incidence in Thailand has risen dramatically, increasing from 112.70 to 146.90 per 100,000 population between 2013 and 2022 [1], primarily due to the aging population. While surgical intervention is crucial [2], recovery presents challenges, with only 12 % of Thai patients walking independently after hip fracture [3]. Mobility decline leads to increased dependence, disability, hospital admissions, institutionalization, and mortality [4], highlighting the urgent need for comprehensive strategies to address this growing public health issue.

Accurate mobility assessment is crucial. However, a lack of standardization in assessment tools creates challenges for nurses. A systematic review [5] identified 19 different instruments, including the Parker Mobility Scale (PMS) [6], the New Mobility Scale (NMS) [7], the 10-m Walk Test (10MWT) [8], and many others [[9], [10], [11], [12], [13], [14], [15], [16], [17], [18], [19], [20], [21], [22], [23], [24], [25]], but no clear “gold standard” exists. This diversity makes it difficult for nurses to choose the most appropriate tool for individual patients, impacting effective care.

The de Morton Mobility Index (DEMMI) is a widely used tool for assessing mobility in diverse populations, including older adults and individuals with acute or chronic medical conditions such as chronic obstructive pulmonary disease, neurological disorders, and hip fractures [15,[26], [27], [28]]. Developed in 2008 [15], the DEMMI consists of 15 items evaluating bedside mobility, seated mobility, gait, dynamic balance, and strength. It has demonstrated strong psychometric properties [[26], [27], [28]]. The DEMMI strongly correlates with established mobility scales for convergent validity, such as the 6-min walk test (*r* = 0.76) and the Barthel Index (*r* = 0.60). Concerning discriminant validity, it maintains low correlations with other measures (*r* = 0.04 to 0.25), indicating it assesses distinct aspects of mobility. The DEMMI also exhibits high internal consistency (minimal detectable change at 90% confidence interval [MDC90] = 8.90), intraclass correlation coefficient (ICC = 0.95), and excellent test-retest reliability (Pearson’s *r* = 0.87). The DEMMI has been translated into multiple languages, including German, Brazilian Portuguese, and Danish [[26], [27], [28]].

While instruments such as the NMS [7], PMS [6], and Cumulated Ambulation Score (CAS) [14] provide basic mobility assessments, they lack the comprehensiveness of tools like the Hip Dysfunction and Osteoarthritis Outcome Score, Joint Replacement (HOOS JR) and the DEMMI [[29], [30], [31]]. HOOS JR and DEMMI offer a more holistic evaluation, addressing symptoms, function, quality of life, mobility, balance, and endurance [[29], [30], [31]]. However, these more comprehensive instruments require more time to administer than NMS, CAS, and PMS.

However, limited research has utilized the DEMMI in Thai patients. The DEMMI, adapted to assess mobility in Thai patients following hip surgery, was published in 2022 [31]. While this initial study provided a foundation, it lacks detailed information on the translation process and its measurement properties. Therefore, this study aimed to translate the DEMMI into Thai and to evaluate its content, convergent, known-group, construct validity, and internal consistency in Thai-speaking patients following hip surgery. Rasch analysis was also employed to examine the psychometric properties of the Thai DEMMI.

## 2 Methods

### 2.1 Study design

This study employed a descriptive-analytical, cross-sectional design to evaluate the validity and reliability of the Thai DEMMI. The study was conducted in two phases. First, in the translation phase, the DEMMI was translated into Thai using a rigorous cross-cultural translation technique [32]. This process ensured the linguistic and cultural equivalence of the instrument for the Thai population. Second, in the psychometric evaluation phase, a cross-sectional study was conducted to assess the validity and reliability of the translated DEMMI. Our study adhered to the Consensus-based Standards for the Selection of Health Measurement Instruments (COSMIN) reporting guidelines for studies on measurement properties [33].

### 2.2 Ethical approval of the study

The Ethics Committee Review Boards of four public hospitals—Vajira Hospital (COA. 140/2565), Krathumbaen Hospital (COA No. 009/65), Singburi Hospital (EC. No. 10/2022), and Phramongkutklao Hospital (IRBRTA.1644/2022)—approved the study. Both oral and written informed consent were obtained from participants. We ensured that all data were analyzed and handled with strict confidentiality.

### 2.3 Translation of the DEMMI into Thai

Permission to translate the DEMMI was obtained from the developer. The cross-cultural translation technique [32] was employed, involving five steps. 1) Forward translation: An instructor from Chulalongkorn University’s Faculty of Nursing, fluent in English and Thai, translated the DEMMI into Thai. 2) Review: A third co-author reviewed the Thai version for accuracy and appropriateness. 3) Back translation: A second translator from Chulalongkorn University’s Faculty of Arts, fluent in both English and Thai, who had not seen the original English DEMMI, translated the Thai version back into English. 4) Discrepancy review: The researchers and translators discussed and reviewed any discrepancies or translation errors. 5) Finalization: The translation process was finalized with the number of items and response scale remaining unchanged, ensuring that the Thai DEMMI accurately reflected the original meaning of the DEMMI items. After the translation procedure, the number of items remained the same, and the response scale was not modified. We only adjusted the Thai language to preserve the intended meaning of the original items.

Inter-rater reliability was assessed between two orthopedic nurses. The researchers organized a meeting with the raters to discuss the test instructions before the raters independently performed the DEMMI on a sample of patients following hip surgery. The raters were blinded to each other’s results. A convenience sample of 30 patients, all aged 50 and older, was recruited from those scheduled for clinic visits at the outpatient departments of public hospitals. The inter-rater reliability was 0.96. We also interviewed and collected feedback from the patients regarding their experiences, feelings, pain, and discomfort during the DEMMI assessments. After the inter-rater reliability and interview, there were no major changes in the Thai DEMMI.

### 2.4 Psychometric evaluation phase

#### 2.4.1 Study settings and participants

A four-stage random sampling method was used to obtain a probability sample of Thai patients after hip surgery. We recruited 260 patients from the outpatient clinics of four general hospitals. Inclusion criteria were age 50 or older, experiencing a first hip fracture due to low-energy trauma, having visited a doctor at the outpatient department between the 3rd and 12th month following hip surgery, and being able to communicate in Thai. Exclusion criteria were a history of severe psychotic disorders, such as Alzheimer’s disease, and an inability to walk before the hip fracture.

We determined that the sample size of 260 patients was adequate based on the requirements for the Rasch analysis. A sample size of at least 200 participants is generally suggested, with 250–500 participants preferred for a robust Rasch analysis [34].

#### 2.4.2 Measures

We selected six scales to conduct the survey. All measures used in the study were obtained with permission from the original developers after we contacted them via email.

##### 2.4.2.1 The demographic and clinical-related questionnaire

This questionnaire included participants’ gender, age, marital status, education, living arrangement, causes of hip fracture, fracture type, type of surgery, Charlson’s comorbidity index [35], and cognitive function [36].

##### 2.4.2.2 The Fatigue Severity Scale (FSS)

A nine-item scale was used to assess the severity of fatigue. Patients rated their responses on a 7-point Likert scale, with one denoting “strongly disagree” and seven denoting “strongly agree.” Scores ranged from 9 to 63, with higher scores indicating more severe fatigue. A score of ≥4 was used as the cutoff point for significant fatigue. The FSS was translated from English into Thai using a forward-only translation approach [37]. Cronbach’s *α* coefficient for the FSS in this study was 0.88.

##### 2.4.2.3 The Pittsburgh Sleep Quality Index (PSQI)

Sleep disruptions and quality were evaluated over the past month using the Pittsburgh Sleep Quality Index (PSQI). In their study, Sitasuwan et al. translated the PSQI into Thai using the forward-back translation technique [38]. The PSQI consists of seven components: sleep length, sleep disruption, sleep latency, daytime dysfunction due to tiredness, sleep efficiency, overall sleep quality, and use of sleep medications. Each component was scored between 0 and 3. The total score, calculated by summing the component scores, ranged from 0 to 21. A higher total score indicated poorer sleep quality, with a score of ≥5 as the cutoff criterion for poor sleep quality [38]. The reliability of the PSQI in the current study assessed by Cronbach’s *α* coefficient, was 0.84.

##### 2.4.2.4 The Numeric Rating Scale (NRS)

This scale measured adults’ current pain intensity. Pain intensity was assessed using a single-item NRS (0–10) on a horizontal scale, where 0 represents “no pain” and 10 means “worst imaginable pain.” Patients were instructed to mark the point on the scale that best represented their pain intensity. The NRS is widely used to assess pain across various diseases and age groups, including older adults, due to its simplicity and responsiveness compared to verbal and visual analog rating scales [39]. Pain frequency was assessed with the question: “Within the past week, how often did the pain occur?” In this study, the test-retest reliability for the NRS was 0.89, and for pain frequency, it was 0.90.

##### 2.4.2.5 The Parker Mobility Scale

This scale was developed to measure mobility and functional ability in patients with mobility impairments, including those recovering from hip fractures and other conditions affecting movement. The PMS consists of three items that evaluate different aspects of mobility, such as walking indoors, walking outdoors, and going shopping or visiting family. Each item is scored based on the patient’s ability to perform the task independently or with assistance. The overall score ranges from 0 to 9, with higher scores indicating better mobility and lower scores indicating greater mobility impairment [6]. In this study, Cronbach’s *α* coefficient for the PMS was 0.88.

##### 2.4.2.6 The de Morton Mobility Index

It consists of 15 items that assess hierarchical aspects of mobility, including mobility in bed and chair, ambulating activities, and balance. Each item is rated on a two- or three-point Likert scale: 0 for “unable,” 1 for “able with minimal supervision or assistance,” and 2 for “independent.” The sum of the scores for each item determines the overall mobility score. The raw score ranges from 0 to 19, where 0 represents dependent mobility, and 19 represents independent mobility. According to de Morton et al. the raw DEMMI scores are transformed to a scale ranging from 0 to 100, with 0 indicating complete mobility impairment and 100 indicating full independent mobility [15].

#### 2.4.3 Data collection

After obtaining Institutional Review Board (IRB) approval, the research team sought the cooperation of nurses to identify patients who met the inclusion criteria. Once potential participants were identified, researchers approached each patient individually to explain the study and invite their participation. Interviews were conducted in a quiet, designated room to ensure privacy and minimize distractions. For patients over 80 years old or with cognitive impairments, researchers involved their caregivers to facilitate communication. Caregivers assisted in ensuring the patients understood the questions and provided accurate responses. For self-rated scales, caregivers completed the forms on behalf of the patients. Only the research team evaluated the patients. All participants who agreed to participate signed a consent form. Data collection took place from January to March 2023. The Thai DEMMI was administered to 260 patients following hip surgery. The mean time to complete each DEMMI assessment was 10.2 ± 2.0 min. No adverse events occurred, such as falls, pain, or injury.

#### 2.4.4 Data analysis

An expert panel comprising five clinicians (one physician, two advanced practice nurses, and two nursing professors) with at least 10 years of experience in hip fracture rehabilitation was assembled for content validity. The panel applied their expertise to evaluate the tool’s relevance, clarity, and alignment with the construct using a 4-point Likert scale. The content validity index (CVI) was calculated at both the item level (I-CVI) and scale level (S-CVI), with thresholds of ≥0.78 for I-CVI and ≥0.90 for S-CVI being considered acceptable [40].

Our current study used Jamovi software version 2.5.6 and SPSS version 29.0.1 for Windows (concurrent licensed through Chulalongkorn University) for data analysis. Participant characteristics and item descriptions were summarized using descriptive statistics, including frequency, mean (± *SD*), standard error of the means (SEM), median with interquartile range (IQR), minimum and maximum values, and range of scores. The raw scores of the DEMMI were transformed to a standardized scale of 0–100 [15].

We examined the correlation between the DEMMI and the PMS scores to assess convergent validity. Prior research has established that the PMS has high validity and reliability, and its adaptation in populations after hip surgery has been well-documented [6,41]. We assessed this correlation using Spearman’s correlation coefficient. A high positive correlation is considered to reflect convergent validity. The size of relationships was interpreted as follows: *r* < 0.30 indicated a low association, 0.30 ≤ *r* ≤ 0.50 indicated a moderate relationship, and *r* > 0.50 indicated a strong relationship [42].

Known-group validity was tested by comparing DEMMI scores for persons categorized by age, gender, cognitive function, comorbidity, length of stay, sleep quality, fatigue, and pain [43]. These differences were supported by prior evidence on factors affecting mobility. Age, gender, comorbidity, cognitive function, length of stay, sleep quality, fatigue, and pain significantly contribute to patients after hip surgery [[43], [44], [45], [46]]. We used an independent *t*-test and ANOVA to examine known-group validity.

Construct validity was tested using confirmatory factor analysis to confirm the factor structure of the DEMMI. The DEMMI was hypothesized as a one-factor model. To imply a good fit of the model to the data, the following criteria were used: the result of equation *χ*^2^/*df* < 3, a Comparative Fit Index (CFI) of ≥0.950, Tucker-Lewis Index (TLI) values of ≥0.950, root-mean-square error of approximation (RMSEA) of ≤0.060, and a standardized root-mean-square residual (SRMR) of ≤0.080 [47].

A polytomous Rasch analysis model was used to evaluate the DEMMI’s validity using information-weighted fit means square statistic (Infit MNSQ) and outlier-sensitive fit means square statistic (Outfit MNSQ). These statistics help determine whether items should be deleted, rescored, or reworded to measure the intended construct appropriately. Infit and Outfit MNSQ close to 1 suggests a good fit, while significantly higher or lower values may indicate a misfit. The Wright (person-item) map also provides insight into the relationship between item difficulty and respondent ability [48,49]. We also employed the item hierarchy (the arrangement of items from easiest to most difficult).

Finally, we evaluated person reliability and the item separation index. Person reliability, analogous to Cronbach’s *α* coefficient, indicates how reliably the DEMMI differentiates between individuals with varying mobility levels. It is expressed as a coefficient ranging from 0 to 1, with values closer to 1 indicating higher reliability. A person reliability of ≥ 0.8 and an item separation index of ≥ 2.0 are benchmarks indicating a good level of separation. These values suggest that the DEMMI has strong discriminatory power and can reliably identify differences in mobility levels among patients after hip surgery [48,49].

Additionally, Cronbach’s *α* coefficient was used to evaluate the internal consistency (or item reliability) of the DEMMI. Cronbach’s *α* coefficient was categorized as follows: *α* ≥ 0.9 = excellent, 0.7 ≤ *α* < 0.9 = good, 0.6 ≤ *α* < 0.7 = acceptable, 0.5 ≤ *α* < 0.6 = poor, and *α* < 0.5 = unacceptable [42].

## 3 Results

### 3.1 Participants’ characteristics and distribution of their DEMMI scores

The sample comprised 260 participants, with women accounting for nearly four times as many participants (79.6 %) compared to men (20.4 %). Almost all participants (96.5 %) lived with family members. Approximately 79.2 % of the participants had no formal schooling, and 12.7 % had completed only a bachelor’s degree. All hip fractures were attributed to falls (100 %). (Appendix A).

The distribution of the DEMMI converted scores is roughly symmetrical. We also recorded floor and ceiling effects, whether 15 % or more of the participants obtained the lowest (0) or highest (19) scale score, respectively. In the current study, neither floor nor ceiling effects occurred since no participant scored 0 or 19 (on the DEMMI raw score) or 100 (on the DEMMI converted score).

### 3.2 Validity

#### 3.2.1 Content validity

The overall CVI for the current study was 0.96, with item-level CVIs ranging from 0.80 to 1.00, indicating good content validity.

#### 3.2.2 Convergent validity

The Spearman Correlation Coefficient of the DEMMI with the PMS scores was examined with a value of *r* = 0.761, *P* < 0.001, 95 %*CI*: 0.70, 0.80, indicating an acceptable validity.

#### 3.2.3 Known-group validity

The DEMMI scores differed significantly among participants classified by age. Older participants had lower DEMMI mean scores compared to younger adults (*F* = 28.27, *P* < 0.001). Based on gender, male participants had higher DEMMI scores than females (*t* = 4.22, *P* < 0.001). Concerning cognitive function and comorbidity, participants with impaired cognitive function and multiple comorbidities had lower DEMMI scores (*F* = 63.00, *P* < 0.001 and *F* = 17.55, *P* < 0.001, respectively).

The length of hospital stay can significantly impact mobility in patients recovering from hip surgery. Participants who stayed in the hospital for less than 7 days after hip surgery had higher DEMMI mean scores compared to those who stayed longer (*t* = 4.75, *P* < 0.001). Furthermore, two groups based on self-reported sleep quality were defined: poor and good sleepers. The mean DEMMI scores were higher in good sleepers than in poor sleepers (*t* = 6.42, *P* < 0.001). Participants who reported fatigue also had lower DEMMI mean scores compared to those who reported no fatigue (*t* = 8.40, *P* < 0.001). Finally, pain was identified as one of the predictive factors of mobility among individuals after hip surgery. Participants experiencing pain had lower DEMMI mean scores compared to those who reported less pain (*F* = 21.23, *P* < 0.001) (Table 1).

**Table 1:** Known-group validity of the de Morton Mobility Index (*n* = 260).

Characteristics	*n* (%)	The scores of the de Morton Mobility Index	*F/t*	*P*
Age (years)			28.27	<0.001
50–59	18 (6.90)	62.39 ± 16.54		
60–69	49 (18.80)	59.10 ± 15.05		
70–79	81 (31.20)	46.51 ± 14.86		
≥80	112 (43.10)	40.77 ± 11.36		
Gender			4.22	<0.001
Male	53 (20.38)	55.34 ± 18.02		
Female	207 (79.62)	45.50 ± 14.33		
Cognitive function			63.00	<0.001
Cognitive impairment	114 (43.84)	37.66 ± 10.39		
Suspected cognitive impairment	116 (44.62)	53.79 ± 14.32		
No cognitive impairment	30 (11.54)	60.63 ± 15.15		
Comorbidity			17.55	<0.001
No (0)	45 (17.31)	58.02 ± 15.66		
Mild (1–2)	130 (50.00)	48.83 ± 14.53		
Moderate (3–4)	57 (21.92)	40.98 ± 12.66		
Severs (≥5)	28 (10.77)	37.75 ± 14.88		
Length of hospital stay (days)			4.75	<0.001
1–7	109 (41.92)	52.72 ± 14.99		
>7	151 (58.08)	43.75 ± 15.03		
Sleep quality			6.42	<0.001
Poor sleepers	230 (88.46)	45.42 ± 14.34		
Good sleepers	30 (11.54)	63.53 ± 16.07		
Fatigue			8.40	<0.001
No fatigue	142 (54.62)	54.10 ± 14.28		
Yes	118 (45.38)	39.58 ± 13.37		
Pain			21.23	<0.001
No pain	48 (18.50)	57.19 ± 14.48		
Mild pain	122 (46.90)	50.11 ± 14.66		
Moderate pain	85 (32.70)	38.26 ± 12.63		
Severe pain	5 (1.90)	48.51 ± 15.63		

*Note*: Data are *n* (%) or *Mean* ± *SD*.

#### 3.2.4 Construct validity

The DEMMI was tested by using confirmatory factor analysis. It was seen that the model indicated a good fit to the empirical data (*χ*^2^ [*df* = 4] = 5.101, *P* = 0.2771, *χ*^2^/*df* = 1.275, RMSEA = 0.033, CFI = 0.998, TLI = 0.995, SRMR = 0.016). The factor loading for each factor ranged from 0.699 to 0.784.

### 3.3 Results of the rasch analysis

#### 3.3.1 Validity

Item Fit Statistics were used to identify items that did not align with the model’s expectations. Issues with an item’s wording, relevance, difficulty, redundancy, or unpredictability could be indicated by such misalignment. Table 2 and Appendix B (a) (b) display the INFIT and OUTFIT statistics for all DEMMI items that met the criteria. However, for two items (items 1 and 3), the OUTFIT statistics did not meet the criteria, indicating that the responses did not align well with the expected responses from the Rasch model.

**Table 2:** Infit MNSQ and outfit MNSQ of the Thai DEMMI (*n* = 260).

Items	Infit MNSQ (ZSTD)	Outfit MNSQ (ZSTD)
DEM 1 Perform a bridge	1.025	29.844
DEM 2 Roll onto the side	0.938	0.635
DEM 3 Lie to sit	1.055	2.211
DEM 4 Sit unsupported in a chair	1.061	0.684
DEM 5 Sit to stand from a chair	0.927	0.802
DEM 6 Sit to stand without using arms	0.845	0.629
DEM 7 Stand unsupported	0.734	0.723
DEM 8 Stand feet together	0.768	0.632
DEM 9 Stand on toes	0.937	0.524
DEM 10 Tandem stand	0.990	0.649
DEM 11 Walking distance	1.055	1.002
DEM 12 Walking independence	0.827	0.851
DEM 13 Pickup pen from the floor	0.903	0.649
DEM 14 Walk backwards	1.000	0.755
DEM 15 Jump	0.949	0.186

*Note*: Infit MNSQ = Information-weighted fit means square statistics. Outfit MNSQ = Outlier-sensitive fit means square statistics. ZSTD = Standardized *Z*-score. DEMMI = the de Morton Mobility Index.

The Wright Map Appendix B (c) and (d) show the alignment between item difficulty and respondent ability. As the latent trait level increases, respondents are more likely to endorse higher response categories, indicating a monotonic increase in endorsement.

Item Hierarchy/Item Difficulty Table 3 presents the hierarchical order of items based on their difficulty in the Thai DEMMI for patients following hip surgery. Among the Thai DEMMI, items such as rolling onto the side and sitting unsupported in a chair were the easiest, while jumping was the most challenging. Items with a high positive logit location (e.g., jump, tandem stand, and stand on toes) indicate greater difficulty, whereas items with a negative logit location (e.g., sit unsupported in a chair, perform a bridge, lie to sit, and sit to stand from a chair) are less difficult. Appendix B (c) and (d).

**Table 3:** Comparison of 15 items’ difficulty between the original DEMMI (2013) and the Thai DEMMI (2025) among patients with hip fracture.

Items	Original DEMMI	Thai DEMMI
DEM 1 Perform a bridge	2	2
DEM 2 Roll onto the side	5	1
DEM 3 Lie to sit	7	4
DEM 4 Sit unsupported in a chair	1	1
DEM 5 Sit to stand from a chair	3	5
DEM 6 Sit to stand without using arms	10	12
DEM 7 Stand unsupported	6	6
DEM 8 Stand feet together	8	7
DEM 9 Stand on toes	13	13
DEM 10 Tandem stand	14	14
DEM 11 Walking distance	4	8
DEM 12 Walking independence	11	9
DEM 13 Pickup pen from the floor	12	11
DEM 14 Walk backwards	9	10
DEM 15 Jump	15	15

*Note*: 1 means the easiest item, and 15 means the most difficulty item. DEMMI = the de Morton Mobility Index.

#### 3.3.2 Person reliability and item separation index

The coefficient of person reliability for the DEMMI was 0.91, indicating high reliability. The item separation index of 10.11 was considered excellent, showing that the Thai version of the DEMMI effectively distinguished among different levels of mobility construct [[48], [49]].

### 3.4 Internal consistency reliability

For internal consistency, according to COSMIN guidelines [33], a minimum of 50 participants is considered a ‘good’ sample size for reliability analysis. We included 260 patients who had undergone hip surgery. Cronbach’s α coefficient for the DEMMI was 0.88, indicating good internal consistency [42]. Item-to-total correlations for most items, except for items 1, 2, 4, and 15, were above 0.30, suggesting that these items were reasonably related to the total score of the DEMMI. Furthermore, all items positively correlated with the total score [42] (Appendix C).

## 4 Discussion

This study followed a structured translation process, including forward-backward translation and established guidelines [32]. The research team, consisting of two bilingual experts and five clinicians with expertise in linguistics and post-hip surgery care, ensured translation accuracy. Discrepancies were promptly resolved through meetings. While the English DEMMI has been translated into German and Dutch [[50], [51]], previous studies lacked detailed discussions on the process, likely due to linguistic similarities between these languages. In contrast, translating English into Thai posed greater challenges due to significant differences in grammar, vocabulary, and cultural context. Thailand’s linguistic diversity further complicates translation, necessitating additional validation with diverse subpopulations to ensure accuracy and applicability [52].

The Thai DEMMI demonstrated excellent content validity through a rigorous process. Meticulous translation and evaluation by bilingual, domain-specific experts ensured clarity and relevance. Pilot testing with the target population provided real-world feedback, refining ambiguous items and enhancing cultural and contextual appropriateness. These efforts ensured that the Thai DEMMI accurately measures mobility in hip surgery patients [42].

The Thai DEMMI demonstrated high convergent validity, strongly correlating with the PMS [42]. The DEMMI is a viable alternative or complement to the PMS, enhancing its clinical utility. While the DEMMI objectively assesses mobility across multiple domains—bed mobility, transfers, balance, and walking [15]—the PMS relies on self-reported mobility within and outside the home, emphasizing functional independence and community participation [6]. Combining clinician-assessed performance (DEMMI) with patient perspectives (PMS), both tools offer a more comprehensive evaluation of mobility.

This study examined the known-group validity of the Thai version of the DEMMI by evaluating its ability to differentiate mobility outcomes across various factors that influenced recovery following hip surgery. These factors included age, gender, comorbidity, cognitive function, length of hospital stay, sleep quality, fatigue, and pain, all known to impact mobility and rehabilitation progress [[44], [45], [46]]. The results are aligned with established evidence that mobility declines with *age* due to reduced muscle strength, balance, and endurance [44]. Gender differences in mobility may stem from variations in muscle mass, pre-injury activity levels, and hormonal factors that influence recovery dynamics [44]. Patients with higher comorbidity scored significantly lower on the Thai version of the DEMMI. This reflected the compounded challenges of managing multiple health conditions, which could slow recovery [44]. Furthermore, the DEMMI effectively distinguished between mobility scores of patients with varying levels of cognitive function, emphasizing its ability to capture the impact of cognitive impairments on mobility recovery [44]. Length of hospital stay was another critical factor, with patients discharged earlier showing higher DEMMI scores. This finding supported that shorter hospital stays often indicated less severe conditions and better functional outcomes. Similarly, patients reporting poor sleep quality demonstrated lower DEMMI scores, highlighting the negative effects of inadequate sleep on energy levels and physical performance during recovery [45]. Fatigue also emerged as a significant factor, as patients experiencing greater fatigue exhibited lower DEMMI scores. This underscores the importance of addressing fatigue to optimize rehabilitation outcomes [46]. Lastly, the Thai version of the DEMMI captured mobility limitations associated with pain, with higher pain levels correlating to reduced mobility. This highlighted the tool's sensitivity to the physical and psychological barriers that pain created during recovery [46].

The CFA results suggested a good model fit for the Thai DEMMI, indicating that the items were reasonably well related to one dimension factor. The result was consistent with the underlying theory of the DEMMI [15]. However, good fit indices did not guarantee a perfect model, especially with a small sample size.

A person reliability coefficient of 0.91 in this study confirms the DEMMI’s ability to distinguish subtle mobility differences among individuals. This ensures consistent, accurate assessments across diverse patient populations. Similarly, an item separation index of 10.11 demonstrated the DEMMI’s effectiveness in differentiating mobility levels [49]-.

The Wright map showed a moderate alignment between item difficulty and patient ability, suggesting an appropriate match. However, the Rasch analysis identified items 1 and 3 as too easy, particularly item 1, while item 15 was too difficult for post-hip surgery patients. Since the DEMMI requires sequential item completion, some overlap in activity performance is expected. Despite this, all 15 items should be retained, as infit statistics remained acceptable [49].

In this study, Cronbach’s α coefficient was 0.88, indicating good reliability [42], consistent with previous findings reporting values between 0.83 and 0.94 [[26], [27], [28]]. We also analyzed extreme response distributions (floor and ceiling effects) to ensure that the DEMMI captured the full range of mobility levels. Our results showed no such effects. This might enhance the DEMMI’s generalizability and usability across diverse populations [42].

## 5 Limitations

A concern in this study was ensuring adequate sample sizes, particularly for ANOVA and subgroup analysis [42]. The study’s generalizability is limited since participants were recruited from only four hospitals—two under the Ministry of Public Health, one university-affiliated hospital, and one under the Ministry of Defense.

## 6 Conclusions

The Thai DEMMI demonstrated strong measurement properties, including content validity, convergent validity, known-group validity, construct validity, and reliability in post-hip surgery patients. However, further research is needed. Longitudinal studies should assess its long-term validity and responsiveness throughout rehabilitation. Additionally, it is essential to explore how technology—such as mobile health apps or electronic health records—can enhance its clinical use.

## CRediT authorship contribution statement

**Chanokporn Jitpanya:** Conceptualization, Methodology, Validation, Formal analysis, Investigation, Data curation, Writing – original draft, Writing – review & editing, Project administration. **Surachai Maninet:** Conceptualization, Methodology, Validation, Formal analysis, Investigation, Resources, Data curation, Writing – review & editing. **Chanipa Yoryuenyong:** Conceptualization, Methodology, Validation, Formal analysis, Investigation, Resources, Data curation, Writing – review & editing, Project administration.

## Data availability statement

The datasets generated during and/or analyzed during the current study are available from the corresponding author upon reasonable request.

## Declaration of competing interest

The authors declare there is no conflict of interest.
