# Supplementary material for: Translation, validity, and reliability of the Thai de Morton Mobility Index in patients following hip surgery
Source: Int J Nurs Sci. 2025 Apr 15;12(4):386–92. doi: 10.1016/j.ijnss.2025.04.003 (PMC12332441; doi:10.1016/j.ijnss.2025.04.003)
Supplement: Multimedia component 1 [file mmc1.docx]

Appendix A Characteristics of the participants (*n* = 260)

| Characteristics | *n* (%) |
| --- | --- |
| Age (years) |  |
| 50–59 | 18 (6.90) |
| 60–69 | 49 (18.80) |
| 70–79 | 81 (31.20) |
| ≥ 80 | 112 (43.10) |
| Gender |  |
| Male | 53 (20.38) |
| Female | 207 (79.62) |
| Cognitive function^a^ |  |
| No cognitive impairment | 30 (11.54) |
| (Suspected) cognitive impairment,  information required | 116 (44.62) |
| Cognitive impairment | 114 (43.84) |
| BMI (kg/m^2^) |  |
| ≤ 18.50 (underweight) | 47 (18.10) |
| 18.50–24.99 (healthy) | 155 (59.60) |
| 25.00–29.99 (overweight) | 52 (20.00) |
| 30.00–39.99 (obese) | 6 (2.30) |
| Comorbidity^b^ |  |
| No (0) | 45 (17.31) |
| Mild (1–2) | 130 (50.00) |
| Moderate (3–4) | 57 (21.92) |
| Severs ( ≥ 5 ) | 28 (10.77) |
| Types of fractures |  |
| Intracapsular fracture | 155 (59.60) |
| Extracapsular fracture | 105(40.40) |
| Types of orthopedic surgery |  |
| Total hip arthroplasty | 31 (11.90) |
| Hemi hip arthroplasty | 130 (50.00) |
| Internal fixation | 99 (38.10) |
| Gait aids use |  |
| None | 44 (16.90) |
| Walker | 135 (51.90) |
| Crane or Crush | 72 (27.70) |
| Wheelchair or Bedridden | 9 (3.50) |
| Length of hospital stay (days) |  |
| 1-7 | 109 (41.92) |
| > 7 | 151 (58.08) |

*Note*: ^a^ Measured by the GP COG screening test. ^b^ Measured by the Charlson Comorbidity Index (CCI)

Appendix B Goodness of fit statistics and the wright map for all DEMMI items (*n* = 260)


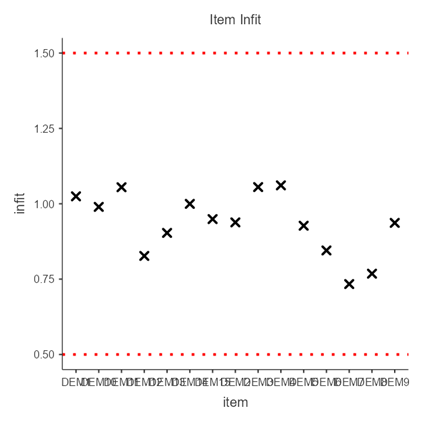

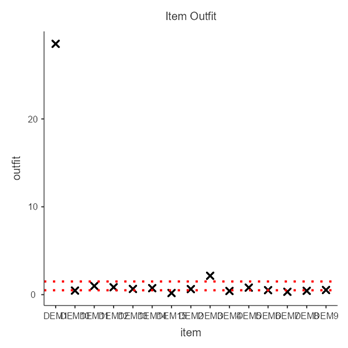


1. (b)


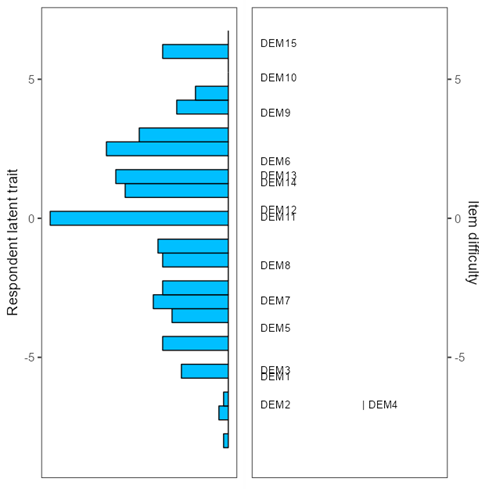

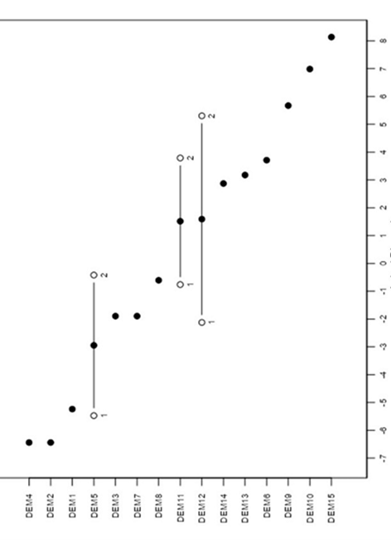


(c) (d)

Appendix C Item reliability analysis (*n* = 260)

| Items | Item-test correlation | If item dropped  Cronbach’s α reliability |
| --- | --- | --- |
| DEM1 | 0.287 | 0.865 |
| DEM2 | 0.246 | 0.867 |
| DEM3 | 0.573 | 0.852 |
| DEM4 | 0.220 | 0.867 |
| DEM5 | 0.691 | 0.845 |
| DEM6 | 0.611 | 0.850 |
| DEM7 | 0.662 | 0.847 |
| DEM8 | 0.723 | 0.842 |
| DEM9 | 0.446 | 0.859 |
| DEM10 | 0.342 | 0.863 |
| DEM12 | 0.735 | 0.842 |
| DEM13 | 0.620 | 0.849 |
| DEM14 | 0.622 | 0.849 |
| DEM15 | 0.247 | 0.866 |
