# Supplementary material for: Translation, validity, and reliability of the Thai de Morton Mobility Index in patients following hip surgery
Source: Int J Nurs Sci. 2025 Apr 15;12(4):386–92. doi: 10.1016/j.ijnss.2025.04.003 (PMC12332441; doi:10.1016/j.ijnss.2025.04.003)
Supplement: Multimedia component 2 [file mmc2.docx]

**泰语版德莫顿活动指数在髋关节术后患者中的验证性分析**

Chanokporn Jitpanya, Surachai Maninet, Chanipa Yoryuenyong

**【摘要】**

**目的** 旨在将德莫顿活动指数（de Morton Mobility Index, DEMMI）翻译为泰语版，并验证其在髋关节术后患者中应用的信效度。

**方法** 采用横断面研究设计。采用正向翻译、审查、反向翻译、差异审查和最终确定5个步骤的跨文化翻译方法，将英文版DEMMI翻译为泰语。于2023 年 1 月至3月，选取泰国4所公立医院的260 例门诊患者为调查对象。采用内容效度、聚合效度、已知组别分析、构念效度评估泰语版DEMMI的效度；Cronbach’s *α*系数评估其信度；Rasch 分析验证其有效性和个人可靠性。

**结果** 泰语版DEMMI条目水平的内容效度为0.80~1.00，量表水平的内容效度为0.96。泰语版DEMMI 与帕克行动能力量表得分存在相关性（*r*=0.761，*P*<0.001）。不同特征患者泰语版DEMMI 得分比较差异具有统计学意义。验证性因子分析结果支持泰语版DEMMI 的假设因子结构，拟合指数良好 [*χ*² (*df* = 4)=5.101，*P*=0.277；*χ*^2^/*df*=1.275，RMSEA=0.033；CFI=0.998；TLI=0.995；SRMR=0.016]。泰语版 DEMMI 具有较高的内部一致性（Cronbach’s *α*=0.88）。Rasch 分析显示良好的个体信度（0.91）和可接受加权的均方拟合统计量值（0.73~1.06）。虽然条目的整体未加权的均方拟合统计量显示出良好的拟合度，但有1个条目的值为29.94，表明存在不拟合现象。**结论** 泰语版 DEMMI 的有效性和可靠性是可以接受的，但仍建议进一步评估其对变化的反应能力。

【**关键词**】髋部骨折；患者；功能恢复；信度；效度

通信作者：Surachai Maninet, E-mail: surachaimaninet@gmail.com
